# Clinical and survival differences between second primary and first primary nasopharyngeal carcinoma in a retrospective study

**DOI:** 10.1007/s12672-025-03250-3

**Published:** 2025-08-12

**Authors:** Xin Huang, Ying Kong, Tianyu Wu, Zhen Meng, Min Kang

**Affiliations:** 1 Department of Radiation Oncology, The First Affiliated Hospital of Guangxi Medical University, Nanning, 530021 Guangxi China; 2 Department of Oncology, Ya’an People’s Hospital, Ya’an, 625000 Sichuan China; 3 Department of Oncology, The Second Affiliated Hospital of Shandong First Medical University, Tai’an, 271000 Shandong China

**Keywords:** Nasopharyngeal carcinoma, Multiple primary cancer, Second primary cancer, Prognosis, Cancer survivors, Survival analysis, Clinical characteristics

## Abstract

**Objective:**

Second primary nasopharyngeal carcinoma (2nd NPC) is defined as nasopharyngeal carcinoma (NPC) diagnosed after another unrelated malignancy. This study aimed to compare clinical profiles, pathological characteristics, treatment patterns, and survival outcomes between patients with 2nd NPC and first primary nasopharyngeal carcinoma (1st NPC).

**Materials and methods:**

We retrospectively analyzed data from patients with multiple primary cancers involving NPC between 2012 and 2023. Patients were classified into 1st NPC (*n* = 103) and 2nd NPC (*n* = 45) groups based on the sequence of NPC diagnosis. Survival and prognostic factors were analyzed using Kaplan-Meier and multivariate Cox regression methods.

**Results:**

The most common extra-nasopharyngeal malignancies in 2nd NPC included breast, colorectal, thyroid, liver, gastric, and bladder cancers. Compared to 1st NPC patients, 2nd NPC patients were significantly older (mean age: 54.0 ± 12.5 vs. 49.5 ± 10.7 years, *p =* 0.027), had higher smoking rates (42.2% vs. 30.1%, *p =* 0.045), and were less likely to present with clinical symptoms (80.0% vs. 97.1%, *p =* 0.001), shorter symptom duration (2.5 vs. 4.0 months, *p* < 0.001), higher comorbidity rates (31.1% vs. 16.5%, *p =* 0.045), and lower Karnofsky Performance Status (KPS ≥ 80: 84.4% vs. 97.1%, *p =* 0.009). Additionally, 2nd NPC patients were more frequently treated with palliative intent (24.4% vs. 8.7%, *p =* 0.010) and showed lower rates of chemotherapy administration (73.3% vs. 89.3%, *p* = 0.014). No significant differences were observed in histologic type, gender distribution, family history, timing of occurrence, interval time, primary tumor site, adjuvant chemotherapy rates, treatment-related toxicity, or treatment intolerance between the groups. However, 2nd NPC was more often diagnosed at earlier stages (stage I/II:17.8% vs. 6.8%, *p* = 0.042). Notably, both overall survival (OS) and progression-free survival (PFS) were significantly shorter in 2nd NPC patients compared to 1st NPC patients (OS: 56.6 months vs. 79.4 months, HR = 1.86, 95% CI: 1.14–3.04, *p* = 0.012; PFS: 46.1 months vs. 74.8 months, HR = 1.98, 95% CI: 1.23–3.12, *p* = 0.0045). Therapeutically, 2nd NPC patients showed significantly lower rates of curative-intent treatment (75.6% vs. 91.3%, *p* = 0.010), lower rates of good treatment tolerance (86.7% vs. 96.1%, *p* = 0.068), reduced chemotherapy utilization (73.3% vs. 89.3%, *p* = 0.014), and less frequent cisplatin use during concurrent chemotherapy (66.7% vs. 84.4%, *p* = 0.034).

**Conclusions:**

Second NPC is not rare. Significant differences in clinical profiles and prognosis between 2nd NPC and 1st NPC, particularly the paradox of earlier-stage diagnosis yet poorer survival and higher risk of disease progression in 2nd NPC, highlight the need for tailored screening, risk-stratified follow-up, and comorbidity-adapted therapies for cancer survivors.

**Supplementary Information:**

The online version contains supplementary material available at 10.1007/s12672-025-03250-3.

## Introduction

Owing to remarkable progress in cancer treatment, the survival rates of cancer patients have significantly improved. However, this success has led to a rapid increase in the prevalence of second primary malignancies (SPMs) [1–4]. SPMs are defined as new malignant tumors that develop either simultaneously or after a primary malignancy, excluding metastasis and recurrence [5]. The occurrence of SPMs can be attributed to multiple factors, including gene mutations, immune dysfunction, exposure to carcinogens (e.g., smoking and alcohol), and the long-term effects of cancer therapies such as radiotherapy and chemotherapy [1, 6, 7]. The prognosis of SPMs is closely related to early detection and timely intervention. Thus, enhancing the diagnosis and treatment of SPMs is of utmost importance for the long-term management of cancer survivors [8, 9].

Nasopharyngeal carcinoma (NPC), an endemic malignancy in Southeast Asia and North Africa [10], can present as either a first primary cancer (1st NPC) or a second primary cancer (2nd NPC). The high incidence of NPC in endemic regions, combined with improved treatment outcomes and prolonged survival, has elevated the risk of 2nd NPC. Regarding SPMs, their risk profiles vary across cancer types. While the development of SPMs may be influenced by shared environmental or genetic determinants and common oncogenic processes, in 2nd NPC, prior cancer-directed therapies—specifically radiotherapy and chemotherapy—represent the principal etiological drivers. These treatments promote carcinogenesis through synergistic genotoxic, inflammatory, and immunosuppressive mechanisms: Radiotherapy causes microhomology-mediated deletions and balanced inversions through misrepair of DNA double-strand breaks, disrupting tumor suppressors such as TP53 in nasopharyngeal epithelial cells [11]. Chemotherapy agents, such as platinum-based drugs and alkylating agents, exacerbate genomic instability via DNA adduct formation and repair inhibition, while therapy-induced cell death releases chromatin fragments that induce inflammation and reactive oxygen species (ROS) [12, 13]. Cisplatin-radiation synergy further impairs DNA repair, and cytokine-mediated immunosuppression (e.g., by TGF-β/IL-6) suppresses cytotoxic T-cell function and compromises immune surveillance of Epstein-Barr virus (EBV)-infected cells. This permits EBV reactivation and expression of the oncoprotein LMP1, which promotes clonal expansion of damaged nasopharyngeal epithelium through NF-κB and PI3K/AKT activation [14–16], ultimately culminating in 2nd NPC.

Despite this established molecular pathogenesis, clinical research on 2nd NPC remains limited, particularly regarding its clinical characteristics, treatment outcomes, and prognosis— especially when compared to the well-studied 1st NPC.Notably, existing studies either focus on malignancies after NPC diagnosis [17–20] or broadly compare NPC patients with vs. without prior cancer history [21–24]. While these studies consistently identify prior cancer as an adverse prognostic factor, direct comparisons between 1st NPC and 2nd NPC as distinct clinicopathological entities are lacking—representing a key gap in current literature.

To address this knowledge gap, we hypothesize that 2nd NPC exhibits poorer prognosis than 1st NPC due to cumulative molecular damage from prior therapies and constrained on treatment options. To test this hypothesis, we conducted a retrospective study comparing the clinical manifestations, pathological features, treatment patterns, and prognostic outcomes of 1st NPC and 2nd NPC. By classifying NPC patients into 1st NPC and 2nd NPC groups based on the sequence of NPC occurrence. We aim to provide valuable insights for clinicians in the diagnosis, treatment, and follow-up of NPC patients, especially those at risk of 2nd NPC, and contribute to the overall knowledge of NPC in the context of multiple primary cancers (MPC).

## Materials and methods

### Clinical subjects

All eligible NPC patients were consecutively enrolled during the study period. We retrospectively reviewed the medical records of all nasopharyngeal carcinoma (NPC) patients with ICD-10 code C11, who were admitted to the First Affiliated Hospital of Guangxi Medical University between January 2012 and December 2023. No patient was excluded based on surveillance intensity or prior cancer screening status.

The diagnosis of MPC was based on the criteria established by Warren and Gates in 1932.The inclusion criteria were as follows [5]: (1) each tumor had to be pathologically confirmed as malignant; (2) each tumor must possess distinct pathological characteristics; (3) tumors should be located in different organs or, if in the same organ, be non-contiguous.

The exclusion criteria included: (1) Lack of pathological evidence; (2) Recurrence or metastasis of the primary cancer; (3) Incomplete clinical data. The patients in the cohort were classified as having second primary nasopharyngeal carcinoma (2nd NPC) or first primary nasopharyngeal carcinoma (1st NPC) based on the sequence in which NPC was diagnosed among MPC. According to the time interval between the diagnosis of the two primary malignant tumors, they were further categorized into synchronous multiple primary cancers (sMPC ) and metachronous multiple primary cancers (mMPC). An interval of ≤ 6 months between the diagnosis of the two cancers was defined as sMPC, whereas an interval of > 6 months was defined as mMPC [25].This retrospective study was reviewed and approved by the Medical Ethics Committee of the First Affiliated Hospital of Guangxi Medical University (Approval No. 2025-E0266). Since it was a retrospective study, the Ethics Committee of the First Affiliated Hospital of Guangxi Medical University waived the requirement for informed consent.

### Clinical data collection

Finally, according to the inclusion and exclusion criteria, a total of 148 patients were included in the study: 103 in the 1st NPC group and 45 in the 2nd NPC group. We retrospectively collected clinical and demographic data from electronic medical records, with all data points independently verified by two researchers to ensure accuracy. Collected parameters included age, gender, smoking and alcohol history, family history, primary tumor site, histological type, clinical symptoms, symptom duration, KPS scores, medical comorbidities, TNM stage, MPC type, interval time, treatment intent, and treatment details. Primary tumor sites were categorized as superior wall, posterior wall, lateral wall, and overlapping lesions, according to the standard classification [22]. Categorized into head and neck (HN) and non - head and neck (non - HN) groups according to the anatomical location of the first primary cancer.The histological subtypes of NPC were classified according to the World Health Organization (WHO) classification as follows: keratinizing squamous cell carcinoma (WHO I subtype), differentiated non-keratinizing squamous cell carcinoma (WHO II subtype), and undifferentiated non-keratinizing squamous cell carcinoma (WHO III subtype) [26]. Symptoms included nasal obstruction, post-nasal drip with blood, tinnitus, deafness, and cervical lymph node enlargement. Medical comorbidities included hypertension, diabetes, cardiovascular diseases, along with other conditions such as chronic obstructive pulmonary disease (COPD), stroke, and chronic kidney disease. Patients with advanced age, poor performance status (e.g., KPS score < 70), prior treatment limitations, or significant medical comorbidities were unable to tolerate or complete curative-intent treatment and thus received palliative therapy. Treatment tolerance was categorized as good or poor, with poor tolerance defined as either toxicity-attributed (CTCAE v5.0-graded) treatment interruption ≥ 7 consecutive days or toxicity-driven regimen modification (≥ 20% dose reduction or drug substitution), while all other cases not meeting these criteria were classified as good tolerance. This assessment was based on retrospective review of clinical documentation including progress notes, physician orders, nursing records, and radiotherapy logs. All toxicities were graded per CTCAE v5.0 criteria by two independent study researchers. Discordant assessments were resolved through adjudication by a senior oncologist. For patients initially diagnosed at other medical institutions (*n* = 10, 6.8%), minor missing data (< 3% per variable) were handled according to STROBE guidelines [27].The pathological evaluation and diagnosis were independently conducted by two specialized pathologists.

### Treatment

The clinical staging of NPC was determined according to the 2017 eighth edition of the American Joint Committee on Cancer (AJCC) staging system. Based on the TNM classification, patients were categorized into early-stage (stage I–II) and advanced-stage (stage III–IV). Treatment adhered to National Comprehensive Cancer Network (NCCN) guidelines, with treatment intent determined through full department discussion: curative denoting complete delivery of NCCN-concordant regimens or definitive radiotherapy (≥ 66 Gy),

and palliative indicating metastatic disease (M1) or non-adherence to guidelines due to age > 75 years, KPS < 70, comorbidities, or prior treatment limitations.Radiation therapy (IMRT) alone for stage I, platinum-based concurrent chemoradiotherapy (CCRT) for stage II, a combination of induction chemotherapy (IC) followed by platinum-based CCRT for stage III-IVA, and systemic therapy combined with localized treatment for stage IVB. The prescribed radiation doses were as follows: 68–76 Gy/30–33 fractions for the primary nasopharyngeal tumor, 66–70 Gy/30–33 fractions for cervical metastatic lymph nodes, 60–64 Gy/30–33 fractions for high-risk clinical target volume (CTV), and 50–54 Gy/30–33 fractions for low-risk CTV. The dose per fraction ranged from 1.8 to 2.33 Gy. Induction or adjuvant chemotherapy regimens included GP (gemcitabine + cisplatin), TPF (docetaxel + cisplatin + 5-fluorouracil), PF (cisplatin + 5-fluorouracil), and TP (docetaxel + cisplatin). Each regimen was administered for 2–3 cycles at 21-day intervals. Concurrent chemotherapy primarily involved cisplatin or other platinum-based drugs [28].

### Endpoints and follow-up

All enrolled patients were followed up through outpatient reviews, inpatient examinations, and telephone calls until death or the study end date. The primary endpoint was overall survival (OS), defined as the time from the diagnosis of NPC to the date of last follow-up or death. Secondary endpoints included progression-free survival (PFS), defined as the time from the date of diagnosis to the first documentation of disease progression or death, and cancer-specific survival (CSS), defined as the time from the date of diagnosis to the date of death caused by MPC.

### Statistical analysis

Categorical variables were expressed as frequencies and percentages and compared using the chi-square test or Fisher’s exact test. Continuous variables were expressed as mean ± standard deviation (SD) and compared using the Student’s t-test or Mann-Whitney U test, as appropriate. Survival analysis was performed using the Kaplan-Meier method, with differences between groups assessed by the log-rank test. Multivariate Cox regression analysis was used to identify independent prognostic factors for OS, PFS, and CSS. Variables with univariate *p* < 0.05 were considered for inclusion in multivariate Cox regression models, with final selection based on clinical relevance and collinearity assessment. Given the rarity of second primary NPC, the cohort size of 2nd NPC patients (*n* = 45) inherently constrains statistical power to detect small-to-moderate effect sizes. We therefore prioritized clinically meaningful differences and interpreted all results with appropriate caution.All statistical analyses were carried out using SPSS software version 27.0 (IBM Corp., Armonk, NY, USA), and R Statistical Software version 4.4.3 (R Foundation for Statistical Computing, Vienna, Austria; https://www.r-project.org/), with a p-value < 0.05 considered statistically significant.

## Results

### Patient characteristics

The demographic and clinical characteristics of the 2nd NPC and 1st NPC groups are shown in Table 1. The mean age of 2nd NPC patients was significantly older than that of 1st NPC patients (54.0 ± 12.5 years vs. 49.5 ± 10.7 years, *p* = 0.027). Additionally, 2nd NPC patients had a higher rate of smoking (42.2% vs. 30.1%, *p* = 0.045) and a slightly higher rate of family cancer history (22.2% vs. 13.6%, *p* = 0.190), but the latter was not statistically significant. There were no significant differences in sex distribution, alcohol history, or body mass index (BMI) between the two groups.

**Table 1:** Clinical characteristics of patients with second primary nasopharyngeal carcinoma (2nd NPC) and first primary nasopharyngeal carcinoma (1st NPC)

Characteristic		Total(*n* = 148)	1st NPC(*n* = 103)	2nd NPC(*n* = 45)	р value
Gender, n (%)					0.203^a^
	Male	109(73.6)	79(76.7)	30(66.7)	
	Female	39(26.4)	24(23.3)	15(33.3)	
Mean age, years (± *SD*)		50.9(±11.4)	49.5(±10.7)	54.0(±12.5)	**0.027**
Smoking history, n (%)					**0.045** ^**b**^
	Never	98(66.2)	72(69.9)	26(57.8)	
	Active	43(29.1)	29(28.2	14(31.1)	
	Heavy	7(4.7)	2(1.9)	5(11.1)	
Alcohol history, n (%)					0.360^a^
	Never	110(74.3)	76(73.8)	34(75.6)	
	Active	27(18.2)	21(20.4)	6(13.3)	
	Heavy	11(7.4)	6(5.8)	5(11.1)	
Family history of tumor, n (%)					0.190^a^
	No	124(83.8)	89(86.4)	35(77.8)	
	Yes	24(16.2)	14(13.6)	10(22.2)	
Mean BMI, kg/m2 (± *SD*)		21.9 ± 3.2	22.1 ± 3.3	21.5 ± 2.9	0.340
MPC type, n (%)					0.480^a^
	Synchronous	34(23.0)	22(21.4)	12(26.7)	
	metachronous	114(77.0)	81(78.6)	33(73.3)	
					0.368^a^
Interval time, years, n (%)	< 5	103(69.6)	74(71.8)	29(64.4)	
	≥ 5	45(30.4)	29(28.2)	16(35.6)	
Median interval time, months(rang)		36.7(9.3–73.5)	25.3(9.7–73.3)	36.7(4.8–85.4)	0.462
Mean diameter of cervical lymph nodes, cm (± SD)		2.1(± 1.4)	2.0(± 1.2)	2.2(± 1.4)	0.435
Histology type, n (%)					0.844^a^
	II	21(14.2)	15(14.6)	6(13.3)	
	III	127(85.8)	88(85.4)	39(86.7)	
Primary site, n (%)					0.830^b^
	Superior wall	15(10.1)	11(10.7)	4(8.9)	
	Posterior wall	30(20.3)	20(19.4)	10(22.2)	
	Lateral wall	43(29.1)	32(31.1)	11(24.4)	
	Overlapping lesion	60(40.5)	40(38.8)	20(44.4)	
Symptom, n (%)					**0.001** ^**b**^
	No	12(8.1)	3(2.9)	9(20.0)	
	Yes	136(91.9)	100(97.1)	36(80.0)	
T stage, n (%)					0.359^a^
	T1/T2	48(32.4)	31(30.1)	17(37.8)	
	T3/T4	100(67.6)	72(69.9)	28(62.2)	
N stage, n (%)					
	N0/N1	43(29.1)	29(28.2)	14(31.1)	0.716^a^
	N2/N3	105(70.9)	74(71.8)	31(68.9)	
M stage, n (%)					0.585^b^
	M0	144(97.3)	101(98.1)	43(95.6)	
	M1	4(2.7)	2(1.9)	2(4.4)	
TNM stage, n (%)					**0.042** ^**a**^
	I/II	15(10.1)	7(6.8)	8(17.8)	
	III/IV	133(89.9)	96(93.2)	37(82.2)	
Symptom duration, months, median (IQR)		3(1.5-6.0)	4.0(2.1–6.5)	2.5(1-3.8)	**< 0.001**
KPS scores, n (%)					**0.009** ^**b**^
	<80	10(6.8)	3(2.9)	7(15.6)	
	≥ 80	138(93.2)	100(97.1)	38(84.4)	
Medical comorbidities, n (%)					**0.045** ^**a**^
	No	117(79.1)	86(83.5)	31(68.9)	
	Yes	31(20.9)	17(16.5)	14(31.1)	
Treatment completion, n (%)					0.068^b^
	No	10(6.8)	4(3.9)	6(13.3)	
	Yes	138(93.2)	99(96.1)	39(86.7)	

Bold indicates a significant difference among groups with *p* < 0.05

Abbreviations: 1st NPC, first primary nasopharyngeal carcinoma; 2nd NPC, second primary nasopharyngeal carcinoma; BMI, Body Mass Index; MPC, multiple primary caners; KPS, Karnofsky Performance Status

^a^Chi-square test, ^b^Fisher’s exact test

Among 1st NPC patients, 97.1% presented with symptoms such as nasal obstruction, post-nasal drip with blood, tinnitus, deafness, and cervical lymph node enlargement. In contrast, among 2nd NPC patients, 80% showed symptoms, while 20% were asymptomatic and diagnosed incidentally during routine follow-up for their prior malignancies. There was a significant difference between the two groups (*p* = 0.001).

### Origin, staging, and treatment of prior cancers in 2nd NPC

The most common prior cancers in 2nd NPC patients were breast cancer (15.6%), colorectal cancer (13.4%), thyroid cancer (11.1%), and liver cancer, gastric cancer, and bladder cancer, each accounting for 6.7% (Supplemental Table 1). Among these patients, 55.6% of prior cancers were diagnosed at stage I/II, 24.4% of patients had a history of radiotherapy, and 40.0% of patients had a history of chemotherapy (Table 2). Synchronous 2nd NPC (diagnosed within 6 months of the prior malignancy) was observed in 12 patients (26.7%), while metachronous 2nd NPC (diagnosed after 6 months) accounted for 33 patients (73.3%), with no significant difference in MPC type between the groups (*p* = 0.480) (Table 1). When stratified by the anatomical site of the first primary cancer, 12 patients (26.7%) had HN origins while 33 (73.3%) had non-HN origins. Notable treatment pattern differences emerged between these groups: HN-origin patients demonstrated significantly higher radiotherapy exposure (50.0% vs. 15.2%, *p* = 0.044) but substantially lower chemotherapy utilization (8.3% vs. 51.5%, *p* = 0.014), with surgical intervention showing a non-significant trend toward higher application in HN-origin cases (100.0% vs. 72.7%, *p* = 0.086) (Table 2).

**Table 2:** Staging and treatment of prior cancers in 2nd NPC patients (Head and neck vs. Non-head and neck)

Characteristic		Total (*n*=45)	HN(*n*=12)	Non-HN (*n*=33)	р value
TNM stage, n (%)					0.742^b^
	Unknown	2 (4.4%)	0 (0.0%)	2 (6.1%)	
	I/II	25 (55.6%)	6 (50.0%)	19 (57.6%)	
	III/IV	18 (40.0%)	6 (50.0%)	12 (36.3%)	
Surgery, n (%)					0.086^b^
	No	9 (20.0%)	0 (0.0%)	9 (27.3%)	
	Yes	36 (80.0%)	12 (100.0%)	24 (72.7%)	
Radiotherapy, n (%)					**0.044** ^**a**^
	No	34 (75.6%)	6 (50.0%)	28 (84.8%)	
	Yes	11 (24.4%)	6 (50.0%)	5 (15.2%)	
Chemotherapy, n (%)					**0.014** ^**b**^
	No	27 (60.0%)	11 (91.7%)	16 (48.5%)	
	Yes	18 (40.0%)	1 (8.3%)	17 (51.5%)	

Bold indicates a significant difference among groups with *p* < 0.05

Abbreviations: HN, head and neck; Non-HN,Non-head and neck

^a^Chi-square test, ^b^Fisher’s exact test

### Diagnosis, histology and treatment of NPC

All patients underwent nasopharyngeal biopsies, and there was no significant difference in the distribution of histological types between the two groups (*p* = 0.844). However, 2nd NPC patients were more frequently diagnosed at earlier stages (stage I/II: 17.8% vs. 6.8%, *p* = 0.042). No significant differences were observed in MPC type, primary tumor site, T stage, N stage, or M stage between the two groups. The mean diameter of lymph nodes in 2nd NPC patients was slightly larger than in 1st NPC patients (2.2 ± 1.4 cm vs. 2.0 ± 1.2 cm, *p* = 0.435), though this difference was not statistically significant (Table 1).

In terms of functional status, 97.1% of 1st NPC patients had a KPS score ≥ 80, compared to 84.4% of 2nd NPC patients (*p* = 0.009). Medical comorbidities were significantly more prevalent in 2nd NPC patients (31.1% vs. 16.5%, *p* = 0.045) (Table 1). Therapeutically, 2nd NPC patients received significantly lower rates of curative-intent treatment (75.6% vs. 91.3%, *p* = 0.010), reduced chemotherapy administration (73.3% vs. 89.3%, *p* = 0.014), and less frequent cisplatin use during concurrent chemoradiotherapy (66.7% vs. 84.4%, *p* = 0.034). No significant intergroup differences were observed in adjuvant chemotherapy rates, grade ≥ 2 hematologic toxicity, or treatment intolerance (all *p* > 0.05; Table 3).

**Table 3:** Treatment characteristics of patients with second primary nasopharyngeal carcinoma (2nd NPC) and first primary nasopharyngeal carcinoma (1st NPC)

Characteristic		Total(*n*=148)	1st NPC(*n*=103)	2nd NPC(*n*=45)	р value
Treatment intent, n (%)					**0.010** ^**a**^
	Palliative	20(13.5)	9(8.7)	11(24.4)	
	Curative	128(86.5)	94(91.3)	34(75.6)	
Radiotherapy, n (%)					0.369^b^
	No	6(4.1)	3(2.9)	3(6.7)	
	Yes	142(95.9)	100(97.1)	42(93.3)	
Chemotherapy, n (%)					**0.014** ^**a**^
	No	23(15.5)	11(10.7)	12(26.7)	
	Yes	125(84.5)	92(89.3)	33(73.3)	
Concurrent chemotherapy drugs, n (%)					**0.034** ^**b**^
	No	23(15.5)	11(10.7)	12(26.7)	
	Cisplatin	117(79.1)	87(84.4)	30(66.7)	
	other platinum - based drugs	8(5.4)	5(4.9)	3(6.6)	
Grade ≥2 hematologic toxicity, n (%)					0.795^a^
	No	133(89.9)	93(90.3)	40(88.9)	
	Yes	15(10.1)	10(9.7)	5(11.1)	
Adjuvant chemotherapy, n (%)					0.393^b^
	No	132(89.2)	90(87.4)	42(93.3)	
	Yes	16(10.8)	13(12.6)	3(6.7)	
Treatment tolerance, n (%)					0.068^b^
	Poor	10(6.8)	4(3.9)	6(13.3)	
	Good	138(93.2)	99(96.1)	39(86.7)	

Bold indicates a significant difference among groups with *p* < 0.05

Abbreviations: 1st NPC, first primary nasopharyngeal carcinoma; 2nd NPC, second primary nasopharyngeal carcinoma

^a^ Chi-square test, ^b^ Fisher’s exact test

### Survival analysis

The final follow-up was conducted on March 15, 2024, with a median follow-up time of 48.7 months (range: 3.4–141.5 months). Among 2nd NPC patients, local recurrences or distant metastases occurred in 8 patients, with a median progression time of 27.1 months (range: 7.7–126.1 months). At the last follow-up, 21 (46.7%) 2nd NPC patients were alive, and 24 (53.3%) had died. Among those who had died, non-nasopharyngeal malignancies were the cause in 4 patients (16.7%). The median overall survival (OS) for 2nd NPC patients was 56.6 months (95% CI: 35.16–78.11).

In contrast, among 1st NPC patients, local recurrences or distant metastases occurred in 15 patients, with a median progression time of 53.4 months (range: 3.4–141.5 months). In the 1st NPC group, 49 patients (47.6%) were alive at the last follow-up, while 54 (52.4%) had died, with 51 deaths due to NPC and 3 to other causes.The median OS for 1st NPC patients was 79.4 months (95% CI: 60.79–97.95).

**Fig. 1:**
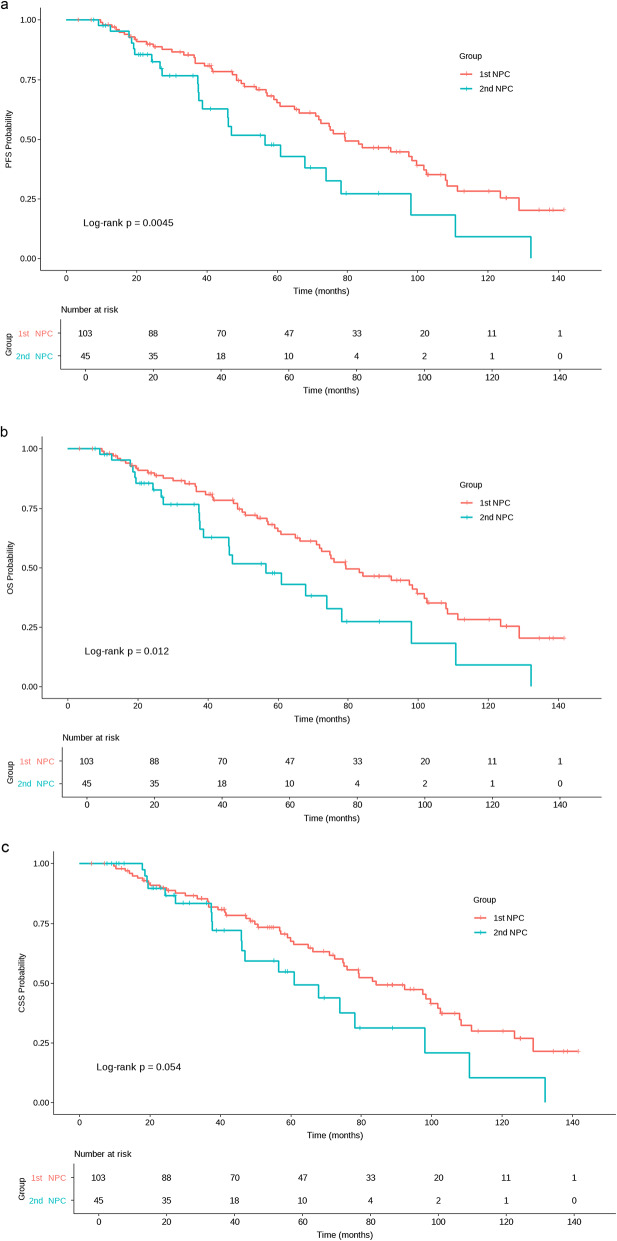
Kaplan-Meier curves for progression-free survival (PFS) **a**, overall survival (OS) **b** and cancer-specific survival (CSS)(c)in patients with first primary nasopharyngeal carcinoma (1st NPC) and second primary nasopharyngeal carcinoma (2nd NPC). Abbreviations: CI, confidence interval; HR, hazard ratio; OS, overall survival; PFS, progression-free survival; CSS, cancer-specific survival;1st NPC, first primary nasopharyngeal carcinoma; 2nd NPC, second primary nasopharyngeal carcinoma. 1st NPC patients had significantly longer PFS (74.8 months vs. 46.1 months; HR = 1.98, 95% CI: 1.23–3.12, *p* = 0.0045) and OS (79.4 months vs. 56.6 months, HR = 1.86, 95% CI: 1.14–3.04, *p* = 0.012) compared to 2nd NPC patients, indicating 1st NPC patients may have a better prognosis

Both OS and progression-free survival (PFS) were significantly shorter in 2nd NPC patients compared to 1st NPC patients.The median PFS for 2nd NPC was 46.1 months (95% CI: 34.85–57.24 months), shorter than 74.8 months (95% CI: 62.15–87.45) in 1st NPC (HR = 1.98, 95% CI: 1.23–3.12, *p* = 0.0045). Notably, the 95% CI width for 2nd NPC PFS (22.39 months) reflects statistical uncertainty from the small cohort (*n* = 45). Similarly, the median OS for 2nd NPC was 56.6 months (95% CI: 35.16–78.11), versus 79.4 months (95% CI: 60.79–97.95) in 1st NPC (HR = 1.86, 95% CI: 1.14–3.04, *p* = 0.012). However, there was no significant difference in CSS between the two groups (*p* = 0.054). Kaplan-Meier survival curves for OS, PFS, and CSS are shown in Fig. 1.

### Prognostic analysis

**Fig. 2:**
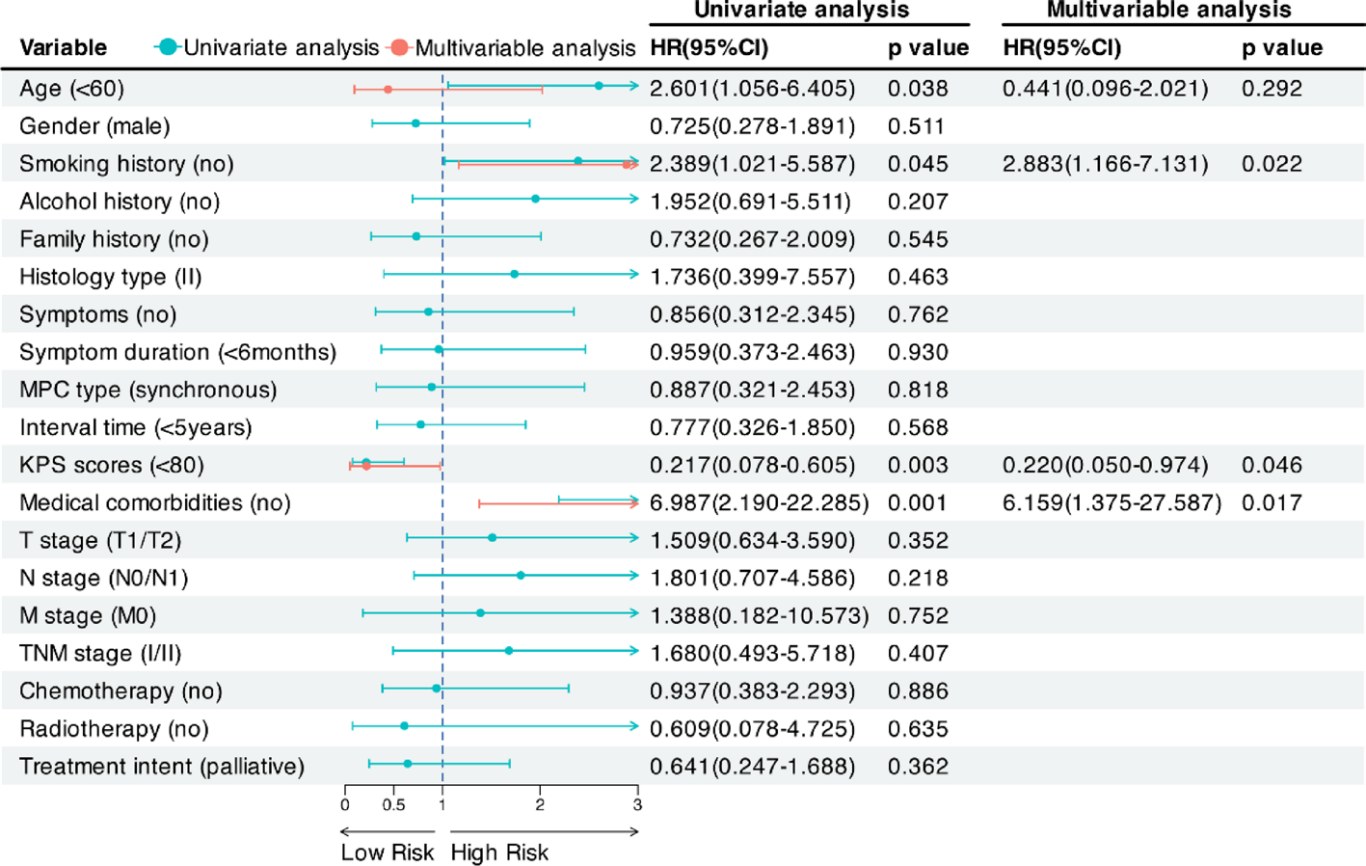
Forest plot of univariate and multivariable survival analyses for overall survival in 2nd NPC patients. Abbreviations: CI, confidence interval; HR, hazard ratio; MPC, multiple primary cancers; KPS, Karnofsky Performance Status; 2ndNPC, second primary nasopharyngeal carcinoma; HR, hazard ratio

Prognostic factors for overall survival (OS) were analyzed using univariate and multivariate Cox regression models. For 2nd NPC patients, univariate analysis identified age, smoking, KPS scores, and medical comorbidities as significant prognostic factors for OS (Fig. 2). Variables significant in univariate analysis (*p* < 0.05) were included in multivariate models, with selection adjusted for clinical relevance (see Methods). After adjusting for potential confounding factors, multivariate analysis revealed that smoking was significantly associated with reduced overall survival (OS) (HR = 2.88, 95% CI: 1.17–7.13, *p* = 0.022), indicating a promoting effect on poor survival. Similarly, the presence of comorbidities was significantly associated with worse overall survival (HR = 6.16, 95% CI: 1.38–27.59, *p* = 0.017), suggesting a strong negative impact on survival. In contrast, KPS ≥ 80 was associated with better overall survival (HR = 0.22, 95% CI: 0.05–0.97, *p* = 0.046), indicating a protective effect.

**Fig. 3:**
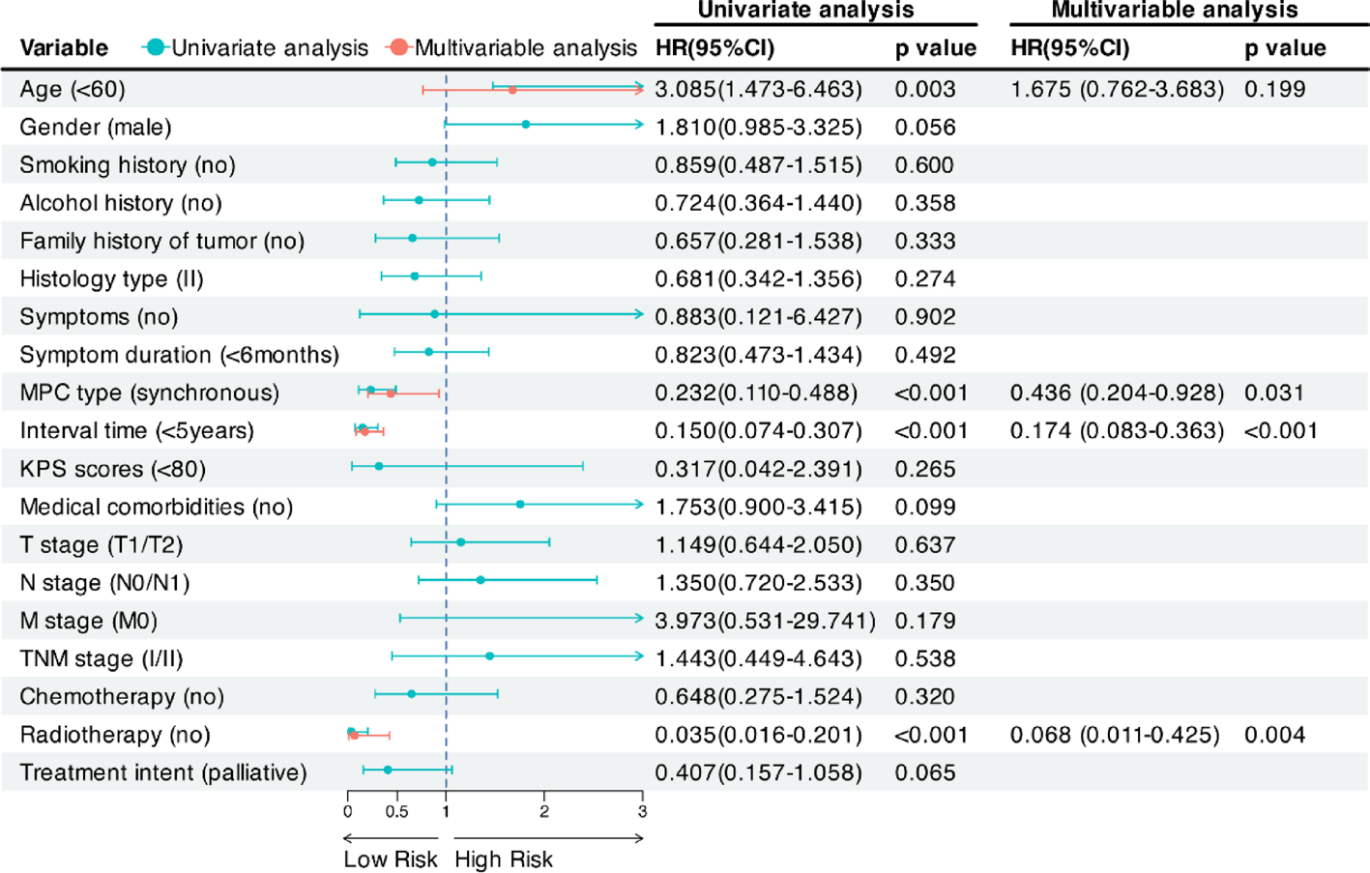
Forest plot of univariate and multivariable survival analyses for overall survival in 1st NPC patients. Abbreviations: CI, confidence interval; HR, hazard ratio; MPC, multiple primary cancers; KPS, Karnofsky Performance Status; 1st NPC, first primary nasopharyngeal carcinoma; HR, hazard ratio

For 1st NPC patients, univariate analysis showed that age, MPC type, interval time, and radiotherapy were significant prognostic factors for OS (Fig. 3). After adjusting for potential confounding factors, multivariate analysis demonstrated that longer interval time (HR = 0.17, 95% CI: 0.08–0.36, *p* < 0.001), radiotherapy (HR = 0.07, 95% CI: 0.01–0.43, *p* = 0.004), and mMPC (HR = 0.44, 95% CI: 0.20–0.93, *p* = 0.031) were associated with better OS, indicating protective effects.

## Discussion

Previous literature has primarily focused on second primary cancers(SPMs) following NPC [17–19]. There have been few studies that investigate NPC as a subsequent cancer occurring after other primary malignancies [21–24]. To our knowledge, no literature has compared the distinctions between the 2nd NPC and the 1st NPC. Thus, our study aimed to compare the clinical features, diagnosis, treatment modalities, and prognoses of 2nd and 1st NPC.

Unhealthy lifestyle habits, such as smoking and excessive alcohol consumption, serve as high-risk factors for various malignancies [1, 4, 6, 7, 29]. In our study, the proportion of smokers was significantly higher among 2nd NPC patients compared to 1st NPC patients (42.2% vs. 30.1%, *p* = 0.045), which is consistent with the findings of Chen et al. [9]. Prior studies have also revealed that persistent smoking is regarded as one of the most potent negative predictors of outcomes in cancer survivors [30]. Conversely, smoking cessation after an initial cancer diagnosis has been shown to correlate with delayed the onset of new malignancies and improve overall survival [30–32]. Hence, this offers evidence-based support for clinicians to encourage patients, particularly cancer survivors, to quit smoking.

Both obesity and a family history of cancer are well-established risk factors for many malignancies [33–35]. Unlike smoking, our study found no significant differences in BMI values or family cancer history backgrounds between 2nd and 1st NPC patients.This suggests that smoking may pose a greater risk factor for the development of 2nd NPC than either obesity or a family history of cancer. Further research is needed to explore whether weight management, combined with genetic counseling and surveillance strategies for those with a family history of cancer, could effectively reduce the risk of SPMs.

Consistent with other SPMs studies [21, 36], our findings indicate that patients diagnosed with 2nd NPC were significantly older than those with 1st NPC (54.0 years vs. 49.5 years, *p* = 0.027). This observation aligns with previous reports, including Wang et al. [23], who noted that NPC patients with a prior cancer diagnosis were approximately 5 years older than those without one. The age difference at diagnosis may be attributed to the fact that patients with the first primary cancer have survived long enough to develop another primary cancer, as well as the cumulative effects of prior treatments such as chemotherapy and radiotherapy [23, 24]. These findings underscore the importance of long-term surveillance for cancer survivors, particularly those who have undergone intensive treatment, to monitor for the potential development of SPMs.

Among 2nd NPC cases, 64.4% occurred within 5 years of the initial cancer diagnosis, which aligns with previous findings. The proportion of synchronous 2nd NPC was 26.7%, higher than the 21.4% observed in the 1st NPC group. Additionally, 35.6% of 2nd NPC cases were diagnosed over 5 years after the first primary malignancy, compared to 28.2% in the 1st NPC group. Although this difference was not statistically significant, it highlights the critical need for comprehensive systemic evaluations before initiating NPC treatment to minimize the risk of overlooking concurrent malignancies at other sites. Moreover, long-term follow-up is crucial not only to monitor primary cancer recurrence and metastasis but also to detect the potential development of secondary primary NPC.

Patients with prior non-nasopharyngeal malignancies tend to receive more frequent imaging examinations, which may facilitate early detection and fewer symptoms of second primary cancers. Our research supports this point: 20% of 2nd NPC patients were asymptomatic at diagnosis, significantly higher than the 2.9% observed in 1st NPC patients (*p* = 0.001), though lower than the 60.0% reported in kidney cancer by Ljungberg et al. [37].This likely contributes to the higher proportion of early-stage diagnoses in 2nd NPC patients (stage I/II: 17.8% vs. 6.8%, *p* = 0.042), as frequent imaging surveillance facilitates earlier detection, often during the asymptomatic phase, compared to 1st NPC cases. However, despite earlier-stage diagnosis, our study revealed that both OS and PFS were significantly shorter in 2nd NPC patients compared to 1st NPC patients (OS: 56.6 months vs. 79.4 months, *p* = 0.012; PFS: 46.1 months vs. 74.8 months, *p* = 0.0045). This finding is consistent with previous studies reporting shorter OS in NPC patients with a history of prior cancer [23, 24], and further extend these findings by demonstrating a significant reduction in PFS as well. Although intensified surveillance in cancer survivors could contribute to earlier-stage diagnosis in 2nd NPC (stage I/II: 17.8% vs. 6.8%), three findings argue against this as the primary driver: First, 77.8% of asymptomatic detections were incidental during non-targeted evaluations; Second, diagnostic timelines for symptomatic patients showed no delay; Crucially, stage-adjusted multivariate analysis confirmed a persistent survival disadvantage for 2nd NPC (OS HR = 1.72, 95% CI:1.08–2.74; PFS HR = 1.85, 95% CI:1.15–-2.98). This suggests the survival disparity reflects biological aggressiveness and/or treatment constraints rather than surveillance artifact.

The prognostic factors for 1st NPC include age, sex, tumor stage, and EBV-DNA [18, 20]. In our study, univariate analysis revealed that for patients with 2nd NPC was significantly influenced by age, smoking status, and KPS, whereas interval time, symptoms, and TNM stage showed no significant association. For 1st NPC patients, significant prognostic factors included age, MPC type, interval time, and radiotherapy, while smoking history, symptoms, and TNM staging did not demonstrate statistical significance. Multivariate analysis further confirmed smoking as an independent prognostic factor for 2nd NPC, consistent with previous literature. Regarding 1st NPC, the analysis revealed that MPC type, interval time, and radiotherapy remained significant determinants of survival outcomes.

The poorer prognosis observed in 2nd NPC patients, as evidenced by significantly shorter OS and PFS, may be attributed to multiple factors, including the cumulative effects of prior cancer treatments, advanced age, and increased medical comorbidities. Prior treatments, such as chemotherapy, radiotherapy, or surgery, which may compromise physiological reserves and limit therapeutic options for subsequent malignancies. For instance, patients with prior head and neck tumors who have received radiotherapy may face dose constraints that restrict additional radiotherapy within a two-year interval due to normal tissue tolerance limits [22, 24]. Similarly, cumulative dose limitations of certain chemotherapeutic agents may preclude their repeated administration, so palliative treatment is chosen, which is potentially linked to incomplete treatment courses [24]. Additionally, advanced age is a well-established risk factor for poorer outcomes in cancer patients. In our study, 2nd NPC patients were significantly older than 1st NPC patients (mean age: 54.0 ± 12.5 vs. 49.5 ± 10.7 years, *p* = 0.027), which may reduce physiological resilience and treatment tolerance. Furthermore, medical comorbidities, which are more prevalent in older patients, are associated with poorer clinical outcomes among cancer survivors [38]. In our cohort, 2nd NPC patients exhibited a significantly higher prevalence of medical comorbidities compared to 1st NPC patients (31.1% vs. 16.5%, *p* = 0.045), further highlighting the impact of comorbidities on prognosis in this population. The combined impact of prior treatments, advanced age, and comorbidities likely contributes to poorer survival in 2nd NPC patients.

Notably, this study found that 2nd NPC was diagnosed at earlier stages (Stage I/II: 17.8% vs. 6.8%, *p* = 0.042) yet demonstrated significantly worse survival than 1st NPC: shorter median overall survival (56.6 vs. 79.4 months, HR = 1.86, 95% CI: 1.14–3.04, *p* = 0.012) and progression-free survival (46.1 vs. 74.8 months, HR = 1.98, 95% CI: 1.23–3.12, *p* = 0.0045). This paradox reflects dual constraints from clinical management limitations and complex tumor biology.

Clinically, prior radiotherapy and chemotherapy imposed critical dose constraints and toxicity thresholds, reducing radical-intent treatment feasibility. Our data confirmed lower utilization in 2nd NPC patients versus 1st NPC controls for radiotherapy (93.3% vs. 97.1%, *p* = 0.369), platinum-based chemotherapy (73.3% vs. 89.3%, *p* = 0.014), and radical therapy (75.6% vs. 91.3%, = 0.010). This aligns with established risk factors—advanced age, comorbidities, and initial treatment limitations [21–24]. Critically, among patients with head and neck primary cancers, 50.0% (6/12) had prior radiotherapy histories—an exposure that constrains re-irradiation due to cumulative organ tolerance, thereby partially explaining the reduced curative-intent treatment rates (75.6% vs. 91.3%). Since radiotherapy is the cornerstone treatment for NPC, exceeding cumulative dose limits directly precludes curative-intent re-irradiation, thereby restricting therapeutic options to palliative approaches and being associated with poor prognosis. Biologically, molecular alterations drove intrinsic resistance: radiotherapy-induced microhomology-mediated TP53 deletions impaired DNA repair, while platinum chemotherapy activated PI3K/AKT/NF-κB pathways to confer cross-resistance [11, 13, 14]. Concurrently, therapy-induced “field cancerization” remodeled the microenvironment: radiation-field mucosal damage promoted HIF-1α-mediated carcinogenesis through chronic hypoxia [39]; epigenetic dysregulation reactivated EBV latency pools; and TGF-β/IL-6 secretion exhausted CD8⁺ T-cells, synergizing with LMP1/NF-κB-mediated immune evasion [15, 16]. The treatment limitations observed in head-and-neck-origin patients with prior radiotherapy validate this theory’s core premise—radiation-remodeled microenvironments compromise subsequent therapy efficacy and accelerate progression. Thus, the convergence of clinical vulnerabilities and molecular resistance mechanisms establishes 2nd NPC as a biologically distinct entity with inherent treatment resistance, explaining its early detection but poor prognosis paradox. These findings underscore the urgency of developing risk-stratified screening, microenvironment-targeted therapies, and comorbidity-adapted regimens to improve outcomes in this high-risk population. Additionally, these observations highlight the necessity for tailored screening, risk-stratified follow-up, and comorbidity-adapted therapies to address the unique challenges and improve outcomes in this high-risk population. The contrast of earlier detection but worse outcomes in 2nd NPC patients underscores the urgency of implementing these strategies to optimize clinical management and survival outcomes.

The poorer prognosis in 2nd NPC patients underscores the need for tailored surveillance strategies. Our findings require cautious extrapolation to non-Asian populations. Given NPC’s endemic predominance in Asia [10, 24, 40], ethnic variations in tumor biology and healthcare disparities may modulate 2nd NPC outcomes in other regions.

## Limitations

This study has several limitations that should be acknowledged. First, the retrospective design may introduce selection bias, particularly through preferential referral of complex cases that could overrepresent aggressive phenotypes in the 2nd NPC group and potentially inflate the observed survival differences. Second, while the cohort size of 2nd NPC (*n* = 45) reflects the real-world rarity of this condition, it substantially increases the risk of type II errors (i.e., failing to detect true differences) and limits subgroup analyses. This constraint was mitigated by our focus on clinically significant outcomes.Third, the unavailability of EBV-DNA data — a well-established prognostic biomarker in NPC—precluded its incorporation into survival analyses, potentially confounding outcome interpretations. Fourth, the relatively short follow-up period may compromise the accuracy of survival data analysis, potentially missing long-term events such as late recurrences or secondary malignancies, which could lead to an incomplete understanding of survival patterns. Fifth, unmeasured residual confounders (e.g., detailed prior treatment toxicities) may persist despite multivariate adjustments. Sixth, the single-center cohort from an NPC-endemic region limits generalizability to non-endemic populations. Finally, the lack of molecular data hinders validation of the proposed biological mechanisms.Future prospective studies with larger cohorts and extended follow-up periods are needed to validate these findings and provide more robust evidence.

## Conclusion

In conclusion, our study demonstrates that 2nd NPC represents a distinct clinical subgroup with unique challenges and poorer outcomes compared to 1st NPC. The unexpected finding of early detection yet poorer survival in 2nd NPC patients suggests the need for tailored screening, risk-stratified follow-up, and comorbidity-adapted therapies. By addressing these challenges, clinicians may improve the long-term management and outcomes of cancer survivors at risk for SPMs. Further studies should elucidate the molecular and genetic mechanisms underlying 2nd NPC and validate the proposed biological pathways to develop targeted interventions for this high-risk population.

## Supplementary Information

Below is the link to the electronic supplementary material.

Supplementary Material 1

## Data Availability

The datasets are available from the corresponding author on reasonable request.
