# Supplementary material for: Clinical and survival differences between second primary and first primary nasopharyngeal carcinoma in a retrospective study
Source: Discov Oncol. 2025 Aug 12;16:1538. doi: 10.1007/s12672-025-03250-3 (PMC12343383; doi:10.1007/s12672-025-03250-3)
Supplement: Supplementary file 1 — Supplementary Material 1 [file 12672_2025_3250_MOESM1_ESM.docx]

Supplemental Table 1 Origin of prior cancers in 2nd NPC

| Location | n (%) |  | Location | n (%) |
| --- | --- | --- | --- | --- |
| Orbit | 1(2.2) |  | Gastric | 3(6.7) |
| Nasal cavity | 1(2.2) |  | Appendix | 1(2.2) |
| Hard palate | 1(2.2) |  | Colorectal | 6(13.4) |
| Tongue | 1(2.2) |  | Uterine cervix | 2(4.4) |
| Hypopharynx | 1(2.2) |  | Kidney | 1(2.2) |
| Larynx | 2(4.4) |  | Urinary bladder | 3(6.7) |
| Thyroid gland | 5(11.1) |  | Prostate | 1(2.2) |
| Breast | 7(15.6) |  | Leukemia | 2(4.4) |
| Lung | 2(4.4) |  | Myeloma | 2(4.4) |
| Liver | 3(6.7) |  | **Total** | 45(100.0) |
|  |  |  |  |  |

Supplemental Table 2 Univariate and multivariable Cox regression analyses for overall survival in patients with second primary nasopharyngeal carcinoma (2nd NPC)

| Variable | Univariate analysis |  | Multivariable analysis |  |
| --- | --- | --- | --- | --- |
|  | HR (95% CI) | *p* value | HR (95% CI) | *p* value |
| Age (<60) | 2.601（1.056-6.405） | **0.038** | 0.441(0.096-2.021) | 0.292 |
| Gender (male) | 0.725（0.278-1.891） | 0.511 | NA | NA |
| Smoking history (no) | 2.389(1.021-5.587) | **0.045** | 2.883（1.166-7.131） | **0.022** |
| Alcohol history (no) | 1.952(0.691-5.511) | 0.207 | NA | NA |
| Family history (no) | 0.732(0.267-2.009) | 0.545 | NA | NA |
| Histology type (II) | 1.736(0.399-7.557) | 0.463 | NA | NA |
| Symptoms (no) | 0.856(0.312-2.345) | 0.762 | NA | NA |
| Symptom duration（＜6months） | 0.959(0.373-2.463) | 0.930 | NA | NA |
| MPC type (synchronous) | 0.887(0.321-2.453) | 0.818 | NA | NA |
| Interval time (＜5years) | 0.777(0.326-1.850) | 0.568 | NA | NA |
| KPS scores (<80) | 0.217(0.078-0.605） | **0.003** | 0.220（0.050-0.974） | **0.046** |
| Medical comorbidities (no) | 6.987（2.190-22.285） | **0.001** | 6.159(1.375-27.587) | **0.017** |
| T stage (T1/T2) | 1.509(0.634-3.590) | 0.352 | NA | NA |
| N stage (N0/N1) | 1.801(0.707-4.586) | 0.218 | NA | NA |
| M stage (M0) | 1.388(0.182-10.573) | 0.752 | NA | NA |
| TNM stage (I/II) | 1.680(0.493-5.718) | 0.407 | NA | NA |
| Chemotherapy (no) | 0.937(0.383-2.293) | 0.886 | NA | NA |
| Radiotherapy (no) | 0.609(0.078-4.725) | 0.635 | NA | NA |
| Treatment intent (palliative) | 0.641(0.247-1.688) | 0.362 | NA | NA |

Notes: Bold indicates statistically significant with *p* < 0.05

Abbreviations: CI, confidence interval; HR, hazard ratio; MPC, multiple primary cancers; KPS, Karnofsky Performance Status; 2nd NPC, second primary nasopharyngeal carcinoma

Supplemental Table 3 Univariate and multivariable Cox regression analyses for overall survival in patients with first primary nasopharyngeal carcinoma (1st NPC)

| Variable | Univariate analysis |  | Multivariable analysis |  |
| --- | --- | --- | --- | --- |
|  | HR(95% CI) | *p* value | HR(95% CI) | *p* value |
| Age (<60) | 3.085(1.473-6.463) | **0.003** | 1.675（0.762-3.683） | 0.199 |
| Gender (male) | 1.810(0.985-3.325) | 0.056 | NA | NA |
| Smoking history (no) | 0.859(0.487-1.515) | 0.600 | NA | NA |
| Alcohol history (no) | 0.724(0.364-1.441) | 0.358 | NA | NA |
| Family history of tumor (no) | 0.657(0.281-1.538) | 0.333 | NA | NA |
| Histology type (II) | 0.681(0.342-1.356) | 0.274 | NA | NA |
| Symptoms (no) | 0.883(0.121-6.427) | 0.902 | NA | NA |
| Symptom duration（<6months） | 0.823(0.473-1.434) | 0.492 | NA | NA |
| MPC type (synchronous) | 0.232（0.110-0.488） | **<0.001** | 0.436(0.204-0.928) | **0.031** |
| Interval time (<5years) | 0.150(0.074-0.307) | **<0.001** | 0.174(0.083-0.363) | **< 0.001** |
| KPS scores (<80) | 0.317(0.042-2.391) | 0.265 | NA | NA |
| Medical comorbidities (no) | 1.753（0.900-3.415） | 0.099 | NA | NA |
| Tstage (T1/T2) | 1.149（0.644-2.050） | 0.637 | NA | NA |
| N stage (N0/N1) | 1.350(0.720-2.533) | 0.350 | NA | NA |
| M stage (M0) | 3.973(0.531-29.741) | 0.179 | NA | NA |
| TNM stage (I/II) | 1.443(0.449-4.643) | 0.538 | NA | NA |
| Chemotherapy (no) | 0.648(0.275-1.524) | 0.320 | NA | NA |
| Radiotherapy (no) | 0.035(0.016-0.201) | **<0.001** | 0.068（0.011-0.425） | **0.004** |
| Treatment intent (palliative) | 0.407(0.157-1.058) | 0.065 | NA | NA |

Notes: Bold indicates statistically significant with *p* < 0.05

Abbreviations: CI, confidence interval; HR, hazard ratio; MPC, multiple primary cancers; KPS, Karnofsky Performance Status; 1st NPC, first primary nasopharyngeal carcinoma
